# Translating Analytical Techniques in Geochemistry to Environmental Health

**DOI:** 10.3390/molecules26092821

**Published:** 2021-05-10

**Authors:** Cathleen L. Doherty, Brian T. Buckley

**Affiliations:** Environmental and Occupational Health Sciences Institute, Rutgers University, Piscataway, NJ 08854, USA; bbuckley@eohsi.rutgers.edu

**Keywords:** ICP-MS, lead isotopes, source apportionment, biomonitoring, geochemistry, environmental health

## Abstract

From human health exposure related to environmental contamination to ancient deep-Earth processes related to differentiation of the Earth’s geochemical reservoirs, the adaptability of inductively coupled plasma mass spectrometry (ICP-MS) has proven to be an indispensable standard technique that transcends disciplines. Continued advancements in ICP-MS, including improved auxiliary applications such as laser ablation (LA), ion/liquid chromatography (IC), automated pre-concentration systems (e.g., seaFAST), and improved desolvating nebulizer systems (e.g., Aridus and Apex) have revolutionized our ability to analyze almost any sample matrix with remarkable precision at exceedingly low elemental abundances. The versatility in ICP-MS applications allows for effective interdisciplinary crossover, opening a world of analytical possibilities. In this communication, we discuss the adaptability of geochemical techniques, including sample preparation and analysis, to environmental and biological systems, using Pb isotopes for source apportionment as a primary example.

## 1. Introduction

The adaptability of analytical techniques utilizing ICP-MS, particularly the use of isotopic signatures, has made it an indispensable transdisciplinary research tool. One such example is high-precision measurements of lead isotopic ratios. In 1956, Clair Patterson’s pioneering work used lead isotopes to define the age of the Earth and meteorites. We refer to this as the geochron, a Pb isochron based on meteorite samples with evolved isotopic ratios, produced by the uninterrupted closed-system decay of U and Th over 4.55 × 10^9^ years [1]. Pb isotopes have since been utilized in geological disciplines to answer questions related to the origin of Earth, petrogenesis, heterogeneity of Earth’s reservoirs, paleoclimatology, oceanography, and atmospheric transport. However, due to the pervasive nature of Pb, environmental contamination of samples remains a primary concern in both sample preparation and interpretation of isotopic signatures. By 1965, Patterson had discovered that atmospheric contamination, observed in both deep-ocean sediments and high-altitude snow, had overprinted the isotopic signatures of Pb across the planet. More than 50 years later, the public health significance of Patterson’s findings continues to inform environmental policy today [2], and Pb isotopes are now recognized as an indispensable tool for both solid Earth geochemistry and environmental health.

Our ability to distinguish between terrestrial sources of lead and anthropogenic lead is critically important if we wish to utilize Pb isotopes as both a geochronometer and an isotopic tracer. However, first we must have a basic understanding of the origin of Pb isotope heterogeneity that leads to distinct isotopic ratios. Pb isotopic ratios are independent of total Pb content, thus making them an effective tracer of both geologic processes and environmental contaminants. The reason is the processes that control isotopic heterogeneity: (1) the abundance of the parent isotopes (i.e., ^238^U, ^235^U, ^232^Th), (2) the radioactive decay rate of these isotopes (t_1/2_ = 4.5 Ga, 700 Ma, and 14 Ga, respectively) into the daughter isotopes (^206^Pb, ^207^Pb, and ^208^Pb), and (3) the time since the formation of the precursor materials (e.g., age of the metal ore deposit). These three factors cause the isotope abundances of a single element to fractionate, or change relative to one another, and therefore cause variability in isotopic ratios from one material to the next. By co-analyzing these decay products (^206^Pb, ^207^Pb, and ^208^Pb) relative to a stable isotope (^204^Pb is produced during nucleosynthesis), we are able to provide a highly specific isotopic fingerprint, a previously underutilized approach for environmental source apportionment.

## 2. Applications

By using comparable methodologies for sample preparation and analysis in environmental health, we can expand the breath of research applications across disciplines. For example, geological samples are highly susceptible to environmental contamination, thus necessitating a pre-cleaning step that involves surface leaching of minerals or rock powders. However, for environmental samples (e.g., soil or dust) with surface adsorption of contaminants, leachates might capture the primary contamination source signature. In both scenarios, surface leaching is a necessary step. Sequential leaching, the incremental leaching of a sample, whereby the supernatant is removed and retained before new solvent is added to the sample, can be used to reveal the inherent composition of the material. By utilizing similar sequential leaching techniques, we can measure systematic isotopic variability and distinguish between surficial contamination and the isotopic signature of the terrestrial material [3]. In contrast, sample preparation for biological samples typically requires a total digestion of the sample, which would erase any potential isotopic heterogeneity. The advantage of working with soft tissue and blood samples is that they can be easily and rapidly digested using either microwave or open-vessel methodologies. In addition, internal standardization via isotope dilution or standard addition is straight forward, and can be used to account for matrix effects. However, there are disadvantages to solution analysis, namely the potential to erase sample heterogeneity in biomarkers that may record changes with growth or development (e.g., teeth and bone). Bulk dissolution would have the effect of re-homogenizing growth zones and obscuring the potential timeline of changes in contaminant exposure.

### 2.1. Sample Preparation

One methodology not broadly applied to environmental and biological samples is the chemical separation of isobaric interferences. While some isobaric interferences can be mathematically corrected for (e.g., ^204^Hg using the isotopic abundance of ^202^Hg), many authors recommend matrix separation and pre-concentration for high-precision measurements (e.g., [4,5]). By utilizing clean laboratories (or class 10/100 laminar flow hoods), we can virtually eliminate laboratory contamination and prolong sample handling for chromatographic separation of isotopes of interest.

For example, the application of geochemical methodologies to biological samples has been demonstrated for the preparation of whole blood for Pb isotopic analysis using multi-collector ICP-MS (MC-ICP-MS) [5]. Fe co-precipitation, which is commonly used in geological studies to pre-concentrate a dilute analyte (e.g., rare earth elements in seawater) and separate the matrix, can also be used to separate Pb from a complex biological matrix like blood. The efficacy of this approach has been successfully demonstrated with reproducibility for lead isotope ratios at <0.23% (*n* = 18) and 0.51% difference between replicate analysis via thermal ionization MS (TIMS) and ICP-MS [5]. This rapid, yet robust procedure allows for efficient sample preparation and higher throughput. For both biological and environmental samples, chromatographic separation using AG 1-X8 anion-exchange resin can also be used to isolate and pre-concentrate Pb (e.g., [6,7,8]). Anion-exchange chromatography can vastly improve precision of low-abundance isotopes (i.e., ^204^Pb = 1.4% natural abundance), which are critical in evaluating differences in source signatures. However, sample preparation is not one size fits all, and investigators should carefully consider the composition of the sample matrix, as well as any concomitant elements, before determining the best separation technique.

An often overlooked preparation step in biological samples is the evaporation of acid following bulk digestion. By adding an evaporation step to biological samples under clean-laboratory conditions (followed by a separation step), we can isolate elements of interest from the primary matrix, improve sensitivity, and minimize isobaric interferences. For solution measurements, the use of automated sample preparation systems (e.g., Elemental Scientific (ESI) SeaFAST) has been shown to improve sample throughput, as well as accuracy, using simultaneous matrix separation and analyte pre-concentration [9]. While this is designed for seawater, small-scale systems with integrated chromatography (e.g., ESI prepFAST MC) are worth exploring for large-scale biological sampling. For low-abundance samples such as blood, particularly in children where availability is limited, introducing chromatographically separated samples through desolvating nebulizer systems (e.g., CETAC Aridus II) allows for higher sensitivity with lower sample consumption, and ultimately improved precision. However, special consideration must be taken to monitor and minimize potential memory effects that may overprint isotopic signatures, especially for subsequent analysis of low-abundance samples. In addition, matrix-matched external reference standards should be included alongside sample, when at all possible. For example, National Institute of Standards and Technology Standard Reference Material (NIST SRM) soils 2709 and 2711, as well as the United States Geological Survey (USGS) basaltic standard BCR-2 have well-characterized metals abundances and Pb isotopic characterization [10,11]. However, finding appropriate reference standards remains a challenge for biological samples, in which case an enriched spike should be added to a control sample to evaluate matrix effects and sample recovery.

### 2.2. Biomonitoring Using Laser Ablation ICP-MS

Laser ablation ICP-MS (LA-ICP-MS), on the other hand, has the advantage of being a fast and arguably less destructive method that is capable of spatial resolution down to ~100–200 um for isotopic analyses [12]. Most promising is the ability to capture a time series of exposure in biological samples based on growth zones (e.g., teeth), and geochemical evolution or P-T changes in geologic samples. The primary disadvantage of LA-ICP-MS is lower precision and accuracy when compared to standard solution methods, particularly the inability to accurately quantify metals in biological matrices for which reference standards are not well established. In contrast, in standard ion-exchange chromatography chemistry, Pb can be isolated from the matrix to remove potential isobaric interferences. However, the level of precision necessary to differentiate between environmental sources of contamination is dependent on the isotopic variation. If there are significant differences, then we can sacrifice some degree of precision in favor of rapid, high-resolution analyses. While in situ isotopic studies of biological samples are limited [12,13], the potential for future studies looks extremely promising in mapping sample heterogeneity. For example, Pb isotopic analysis of teeth can be used to trace modern fetal and childhood exposure to lead [14], while secondary teeth can preserve isotopic signatures of geographic location, diet, and health in mummified human remains [15], demonstrating that recent advancements in ICP-MS, specifically the coupling of LA systems, have expanded an entire field of research.

In order to transfer methodologies that utilize LA-ICP-MS, careful consideration must be taken when trying to quantify metals abundances. The use of LA-ICP-MS to quantify metals in teeth and toenails as a biomarker of past chemical exposures has been demonstrated (e.g., [16,17,18,19]), but typically looks at relative changes in abundance with growth. While standard reference materials with trace elements in glass or basaltic glass are well characterized (e.g., NIST SRM 612, USGS BHVO, USGS BCR-2), standardization becomes more difficult when matrix-matched biological samples are not readily available. Therefore, we recommend a multi-step approach to standardization and validation for elemental quantification by LA-ICP-MS in biological samples: (1) sample calibration using well-characterized standard reference materials (e.g., NIST SRM 612 and 614), (2) normalization of trace element abundances to a structurally significant major element (e.g., % CaO in bone or teeth) measured independently by electron microprobe to correct for variations in amount of sample ablated, and (3) routine analysis of a closely matched matrix standard that has been prepared for ablation (e.g., pressed pellet). The use of high-density pressed pellets of NIST SRM 1486 bone meal may be used as a hydroxyapatite matrix-matched reference standard for analysis of teeth [18]. While for other biological samples such as toenails, trace element abundances can be quantified using a pressed pellet of USGS keratin standards (e.g., USGS42, and USGS43). Regardless of standard utilized, the LA-ICP-MS results of reference standards should be cross-calibrated to acid-digested standard in solution. In addition, confirmatory analysis of Pb isotopic signatures in teeth can be similarly performed using high-precision micro-drilling and extraction using a novel laser ablation sampling technique (e.g., [20]). These micro-samples can then be prepared for single-collector ICP-MS (SC-ICP-MS) or MC-ICP-MS analysis using standard acid-digestion techniques and subsequent anion-exchange chromatography, as previously discussed. This allows for comparison between lead isotopic ratios acquired in situ via LA-ICP-MS and in solution via SC-ICP-MS or MC-ICP-MS. Regardless of sample form (aqueous vs. solid), ICP-MS analysis of Pb isotopes remains comparable. Isotopic abundances of ^202^Hg (to correct for ^204^Hg interference), ^203^Tl, ^204^Pb, ^205^Tl, ^206^Pb, ^207^Pb, and ^208^Pb must be co-analyzed. In addition, samples and standards should be background subtracted, mass bias corrected using ^203^Tl/^205^Tl = 0.41892, and bracketed by NIST SRM 981 Pb isotopic standard and normalized to ^206^Pb/^204^Pb = 16.9405, ^207^Pb/^204^Pb = 15.4963, and ^208^Pb/^204^Pb = 36.7219 [21].

However, the main determining factor in whether in situ isotopic analysis via LA-ICPMS is feasible is the abundance of metal. For example, prior Pb biomarker studies have demonstrated a typical range of Pb measured in the teeth of exposed children at 40–5600 ppb [17,22], with higher levels (940–30600 ppb) reported in certain communities (e.g., mining communities; [23]). For samples with <ppm level Pb, the uncertainty increases. Therefore, analysis of more recently shed teeth may be difficult unless there is evidence of chronic exposure that resulted in high Pb incorporation during development.

### 2.3. Source Apportionment

The potential for Pb isotopic ratios in environmental source apportionment has also been demonstrated with promising results [24,25,26,27]. Source apportionment refers to our ability to identify how much a contamination source contributes to the overall pollutant concentration at a particular site or in a biological sample. Pb isotopic analysis of blood has been shown to be influenced by the bioavailability of the exposure source (among other confounders), indicating that the high Pb content sources may not necessarily be the primary source of exposure in an individual [28]. Therefore, case studies that identify exposure sources by Pb content alone are likely to be missing a primary contributor to the Pb exposure. Pb isotopic fingerprinting also helps to elucidate metal content and mobility in diffuse pollution sources in groundwater [29]. High-precision isotopic analysis of environmental Pb sources is the most promising way to identify Pb contamination sources and mitigate human exposure. Previously, a common flaw has been the absence of ^204^Pb analysis. This is due to the difficulty in measuring ^204^Pb at concentrations below the limit of quantification for the available instrumentation (e.g., quadrupole ICP-MS). Subsequently, binary mixing trends observed when looking only at ^207^Pb/^206^Pb versus ^208^Pb/^206^Pb are often the result of U and Th decay of common terrestrial source, creating an inability to distinguish between more than two Pb contaminant sources [30]. Fortunately, improvements in instrumentation (e.g., magnetic sector and MC-ICP-MS) and adoption of geochemical methodologies have allowed us to revisit Pb isotopes as a robust tracer of multiple environmental Pb sources. Advancements in ICP-MS have allowed us to overcome the analytical limitations of isotopic analysis, particularly the use of multi-collector ICP-MS, which allows for the simultaneous acquisition of signal intensities over a narrow mass range (e.g., 204, 206, 207, 208). While high-precision measurements of Pb isotopes using MC-ICP-MS are routine in geochemical research [31], environmental health has been slower to adopt this technology [32]. Magnetic sector ICP-MS is significantly more sensitive because of the higher ion extraction efficiency, and higher-precision isotopic ratios can be achieved through longer acquisition times. In geologic samples, more subtle variations in isotopic ratios require higher-precision measurements with greater sensitivity (e.g., MC-ICP-MS or thermal ionization MS, TIMS). However, isotopic analysis may be possible with single-collector ICP-MS for environmental and biological samples that have significantly different source signatures [28]. The ultimate advantage of using MC-ICP-MS is the significant improvement in stability and precision (e.g., uncertainty on ^206^Pb/^204^Pb for replicates of NIST 981 < 0.01% on the MC-ICP-MS vs. 0.14% on the single collector), reducing the influence of counting statistics.

By co-analyzing three isotopic ratios (^206^Pb/^204^Pb, ^207^Pb/^204^Pb, and ^208^Pb/^204^Pb), we can provide a highly specific isotopic fingerprint as has previously been demonstrated (e.g., [33,34]). For human biomarker studies, the isotopic ratios of Pb can be used to create multi-component mixing models of primary sources, and then used for source apportionment of Pb in biological samples, describing the exposure in an individual.

In order to apportion sources, isotopically distinct sources must be identified and extrapolated to “end-member” type compositions. First, precise isotopic measurements of potential sources (e.g., soil, water, plumbing, paint, and dust) are made, and then evaluated in three-dimensional isotopic space (^206^Pb/^204^Pb vs. ^207^Pb/^204^Pb vs. ^208^Pb/^204^Pb). Finally, we can use the isotope ratios measured in biomarkers, which represent a potential mixture of sources, to calculate the proportion of each source component using Equation 1,

(1) P207b/P204bmix=P207b/P204bACPbAFMA+P207b/P204bBCPbBFMBCPbAFMA+CPbBFMB

where A, B = end-member components, M = mixture, C = Pb concentration, and F = fraction of component.

**Figure 1:**
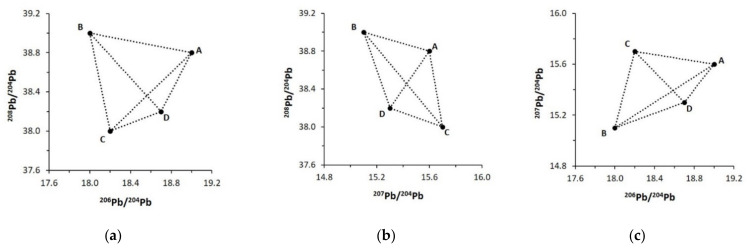
Two-dimensional Pb isotope diagrams for (**a**) ^208^Pb/^204^Pb vs. ^206^Pb/^204^Pb, (**b**) ^208^Pb/^204^Pb vs. ^207^Pb/^204^Pb, and (**c**) ^207^Pb/^204^Pb vs. ^206^Pb/^204^Pb showing the field of mixing for four distinct sources (A, B, C, D).

This approach is typically used to model binary or ternary mixtures, with two or three distinct sources. However, multi-component mixtures become increasingly challenging to resolve when there are more than three primary sources. For example, we demonstrate the field of mixing for four isotopically distinct sources (A, B, C, and D). In standard 2D isotopic diagrams, samples within the field could represent multiple scenarios of mixing, making it difficult to accurately apportion sources (Figure 1).

**Figure 2:**
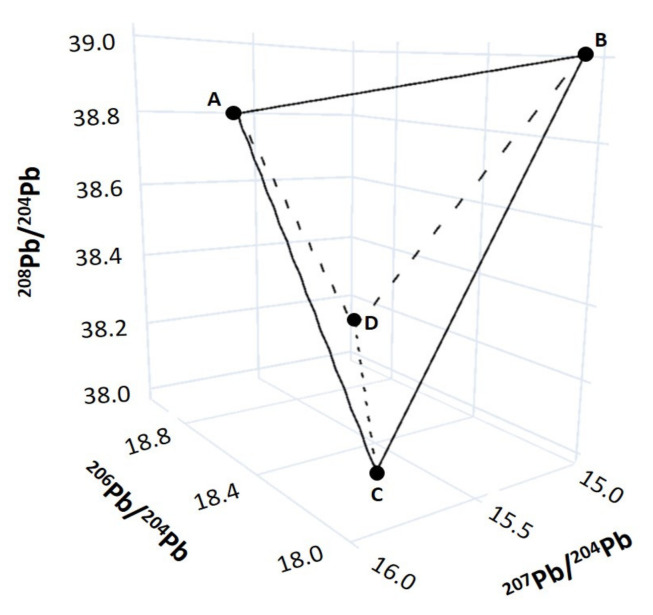
Three-dimensional Pb isotope diagram for ^208^Pb/^204^Pb vs. ^206^Pb/^204^Pb vs. ^207^Pb/^204^Pb showing the field of mixing for four distinct sources (A, B, C, D).

Fortunately, Pb isotopes have three unique isotopic ratios (^206^Pb/^204^Pb, ^207^Pb/^204^Pb, and ^208^Pb/^204^Pb) that can be plotted in 3D space to both visualize isotopic mixtures, and determine whether we have identified the correct sources (Figure 2). Further, Bayesian isotope mixing models, used in conjunction with traditional 3D isotope plots have been shown to improve uncertainty in source apportionment, and more accurately characterize potential sources [35].

The greatest success for source apportioning human exposure comes from identifying and using pure source end-members from primary sources in the home. The advantage of this approach is that we can model multiple scenarios of mixing (binary and ternary) that are unique to the exposed individual. For biological samples where sources cannot be isotopically distinguished, the relative proportions of concomitant metals (e.g., Cd/Pb, Cu/Pb, and Cr/Pb) may reveal primary source signatures [24]. For example, changes in concomitant metals have been shown to indicate dietary changes, as recorded in growth zones of teeth (e.g., changes in Pb, Ba, and Sr) [36,37]. These studies demonstrate the capabilities of both ICP-MS and in situ isotopic analysis via LA-ICP-MS, and our ability to use source apportionment for human exposure would otherwise not be possible without modern advancements to ICP-MS.

## 3. Future Directions

Most recently, advancements in ICP-MS have enabled researchers to understand the behavior of nanoparticles (NPs) in environmental and biological systems. A major challenge in tracing transport and accumulation of NPs in biological systems is the ability to quantify their heterogeneous distribution within an organism, which may be compromised by high background metal levels coupled with low concentration of NPs in a particular organ/system. Stable isotope labeling of engineered NPs produces a unique isotopic fingerprint that can be used to trace the uptake and distribution of metal containing NPs in biological systems [38]. Stable isotope labeling has previously been used to account for analyte transport and loss, as well as in biological uptake studies, but has not been explored for nanoplastics. However, isotopic fractionation remains a concern for many elements, including Fe, Zn, Cu, and Cd, and choice of metal should be carefully considered. Stable labeled NPs using well-characterized isotope systems such as Pb are worth further investigation. Nevertheless, ICP-MS is shown to be the most promising tool for metal-based NP analysis [39].

The use of ICP-MS in in the fields of environmental health and toxicology is rapidly growing, propelled by the adaptability of geochemistry to various sample matrices. These applications extend well beyond Pb isotopic studies and can be utilized for trace element quantitation in biomonitoring studies [40], stable labeled isotopes in engineered nanoparticles [41], enriched isotopes as tracers in biological systems [42], among many other applications. With continued improvements in sensitivity, element selectivity, and low sample consumption, the use of ICP-MS continues to enhance the development of novel techniques to assess environmental exposure and human health.
